# Small HSPs play an important role in crosstalk between HSF-HSP and ROS pathways in heat stress response through transcriptomic analysis in lilies (*Lilium longiflorum*)

**DOI:** 10.1186/s12870-022-03587-9

**Published:** 2022-04-19

**Authors:** Yunzhuan Zhou, Yue Wang, Fuxiang Xu, Cunxu Song, Xi Yang, Zhao Zhang, Mingfang Yi, Nan Ma, Xiaofeng Zhou, Junna He

**Affiliations:** Beijing Key Laboratory of Development and Quality Control of Ornamental Crops, College of Horticulture, China Agricultural University, Beijing, 100193 People’s Republic of China

**Keywords:** *Lilium* sp., Heat stress response, RNA-seq, Heat stress transcription factors, Small heat shock proteins, Reactive oxygen species

## Abstract

**Background:**

High temperature seriously limits the annual production of fresh cut lilies, which is one of the four major cut flowers in the global cut flower market. There were few transcriptomes focused on the gene expression of lilies under heat stress. In order to reveal the potential heat response patterns in bulbous plants and provide important genes for further genetic engineering techniques to improve thermotolerance of lily, RNA sequencing of lilies under heat treatments were conducted.

**Results:**

In this study, seedlings of *Lilium longiflorum* ‘White Heaven’ were heat-treated at 37 °C for different lengths of time (0 h, 0.5 h, 1 h, 3 h, 6 h, and 12 h with a 12 h-light/12 h-dark cycle). The leaves of these lily seedlings were immediately collected after heat treatments and quickly put into liquid nitrogen for RNA sequencing. 109,364,486–171,487,430 clean reads and 55,044 unigenes including 21,608 differentially expressed genes (DEGs) (fold change ≥2) were obtained after heat treatment. The number of DEGs increased sharply during the heat treatments of 0.5 h–1 h and 1 h–3 h compared to that of other periods. Genes of the heat stress transcription factor (HSF) family and the small heat shock proteins (small HSPs, also known as HSP20) family responded to heat stress early and quickly. Compared to that of the calcium signal and hormone pathways, DEGs of the HSF-HSP pathway and reactive oxygen species (ROS) pathway were significantly and highly induced. Moreover, they had the similar expression pattern in response to heat stress. Small HSPs family genes were the major components in the 50 most highly induced genes at each heat stress treatment and involved in ROS pathway in the rapid response to heat stress. Furthermore, the barley stripe mosaic virus induced gene silencing (BSMV-VIGS) of *LlHsfA2* caused a significantly reduced thermotolerance phenotype in *Lilium longiflorum* ‘White Heaven’, meanwhile decreasing the expression of *small HSPs* family genes and increasing the ROS scavenging enzyme *ascorbate peroxidase* (*APX*) genes, indicating the potential interplay between these two pathways.

**Conclusions:**

Based on our transcriptomic analysis, we provide a new finding that small HSPs play important roles in crosstalk between HSF-HSP and ROS pathways in heat stress response of lily, which also supply the groundwork for understanding the mechanism of heat stress in bulbous plants.

**Supplementary Information:**

The online version contains supplementary material available at 10.1186/s12870-022-03587-9.

## Background

The global temperature is increasing, and it has been predicted that the extreme annual daily maximum temperature will increase by about 1 °C to 3 °C by the mid-twenty-first century [1]. A yield loss of 6 to 7% per 1 °C increase in seasonal mean temperature associated with extreme heat disasters has been estimated [2]. High temperature is a major abiotic factor that affects plant growth, and declines yield and quality. Unable to avoid damage from adverse environments through migration as animals do, plants have evolved complex regulatory networks to cope with adverse environments [3, 4]. Four main pathways play important roles in response to heat stress in plants including: the heat stress transcription factor-heat shock protein (HSF-HSP) pathway, calcium ion-calmodulin (Ca^2+^-CaM) pathway, hormone regulatory pathway, and reactive oxygen species (ROS) pathway [4–7].

The primary sensing event of heat stress in plants occurs at the plasma membrane, which leads to the opening of a specific calcium channel. This triggers an influx of calcium ions into the cell as the primary signal that increases the expression of mitogen-activated and calcium-dependent protein kinases, activating the heat stress response (HSR) [6, 8, 9]. During the HSR, hundreds of specific genes, comprising up to 4% of the higher plant genome, are predominantly up-regulated and hundreds of heat shock proteins (HSPs) accumulate in various cellular compartments [6, 10]. HSPs are mainly attributed to the signaling and thermotolerance mechanisms in the HSR, which are well-known targets of HSFs [11]. HSPs are molecular chaperones that belong to several canonical conserved families in prokaryotes and eukaryotes, such as HSP100s, HSP90s, HSP70s, HSP60s, small HSPs, and their co-chaperones [10]. Previous studies have shown that heat signals are probably transduced by several pathways to converge on HSFs, which then activate a number of HSPs and other heat-responsive genes that drive the heat adaptation process in plants [4, 9, 12]. Therefore, the HSF-HSP pathway plays an irreplaceable role as one of the major pathways regulating plant responses to heat stress.

Reactive oxygen species (ROS) play an important role in the heat signaling cascade by regulating the expression and accumulation of stress-response molecules, such as antioxidants, osmotic regulation substances, and HSPs, thereby alleviating heat damage and increasing plant stress tolerance [13]. Additionally, ROS act as a signal to trigger HSR under high temperatures, activating the HSF-HSP pathway [6]. Several studies have also shown that ROS are signaling molecules involved in acquired thermotolerance [14, 15] and their short-term production is key to regulating metabolism in chloroplasts during the acclimation process [16, 17].

Lilies are the multi-annual herb and one of the four major cut flowers in the global cut flower market, which are native to temperate and boreal regions of the Northern Hemisphere and grow well in a cool environment [18, 19]. However, most of them have poor heat tolerance, and their growth and development are inhibited by ambient high temperatures over 30 °C [20, 21]. High environmental temperature seriously limits the annual production of fresh cut lily in many regions. Studies on the mechanisms of heat response in lily will provide candidate regulatory genes for genetic engineering techniques to improve the thermal response of popular lily varieties in market, which will stabilize the annual production of cut flowers in high-temperature areas. In this study, we provided transcriptomic data on the response of lily to heat stress in bulbous plants. The results here indicated that the HSF-HSP pathway and ROS pathway played important roles and have crosstalk through small HSPs family genes under heat stress. These findings provided new meaningful information for understanding the regulation mechanism of HSR in lily and other plants.

## Results

### De novo assembly, assessment and gene annotation of reads

Leaves of aseptically grown seedlings of *L. longiflorum* ‘White Heaven’ were subjected to heat stress under various time durations (0, 0.5, 1, 3, 6, and 12 h) and showed significantly reduced chlorophyll content (Supplementary Fig. 1). They were collected for the extraction of total RNA to perform RNA sequencing. A total of 365.89 Gb of clean data was obtained with a Q30 percentage ranging from 89.52 to 91.42%. 109,816,168–172,328,018 raw sequencing reads were generated and a range of 109,364,486–171,487,430 clean reads were obtained after the quality filtering process (Supplementary Table 1). The clean reads were matched to the assembled sequences with matching ratios ranging from 79.70 to 83.69% (Supplementary Table 1). A total of 55,044 unigenes was obtained with an N50 length of 2249 bp and an average length of 1336 bp (Supplementary Table 2). A total of 31,243 (57%) unigenes was shorter than 1000 bp in length, and 23,801 (43%) unigenes was longer than 1000 bp (Supplementary Table 2). These results indicated that the transcriptome data was high-quality and could provide effective information for heat stress studies.

All the unigenes were selected for functional annotation by searching six public databases as non-redundant protein (NR), protein family (Pfam), Clusters of Orthologous Groups of proteins (COG), Swiss-prot, Gene Ontology (GO) and Kyoto Encyclopedia of Genes and Genomes (KEGG). A total of 32,328 (58.73%) unigenes was successfully annotated based on similarity to sequences in all databases by performing a BLAST search (Table 1). Approximately 31,791 unigenes (57.76%) was annotated using the NR database, a total of 27,773 unigenes (50.46%) were annotated using the COG database and grouped into 23 potential functional classifications (Supplementary Fig. 2). The “replication, recombination, and repair” group (4047; 14.57%) was the largest known group, followed by “posttranslational modification, protein turnover, and chaperones” (2045; 7.37%), “transcription” (1472; 5.30%), and “signal transduction mechanisms” (1173; 4.22%) groups. Notably, there were 368 genes enriched into the term “inorganic ion transport and metabolism” group, which might be related to sensing of heat stress through plasma membrane. In addition, 27,166 unigenes (49.35%) were classified into 49 GO functional terms among three main categories: biological processes, cellular components, and molecular functions (Supplementary Fig. 3). A total of 13,336 unigenes (24.23%) was mapped to 27 KEGG pathways (Supplementary Fig. 4), of which several pathways were enriched, including “metabolism of cofactors and vitamins,” “carbohydrate metabolism,” “folding, sorting and degradation,” “Translation,” and “Signal transduction.”

**Table 1:** Gene function annotation

	Number of annotated unigenes	Number of annotated unigenes
GO	27,166	49.35%
KEGG	13,336	24.23%
COG	27,773	50.46%
NR	31,791	57.76%
Swiss-Prot	22,787	41.40%
Pfam	21,787	39.58%
Total_anno	32,328	58.73%
Total	55,044	100%

**Fig. 1:**
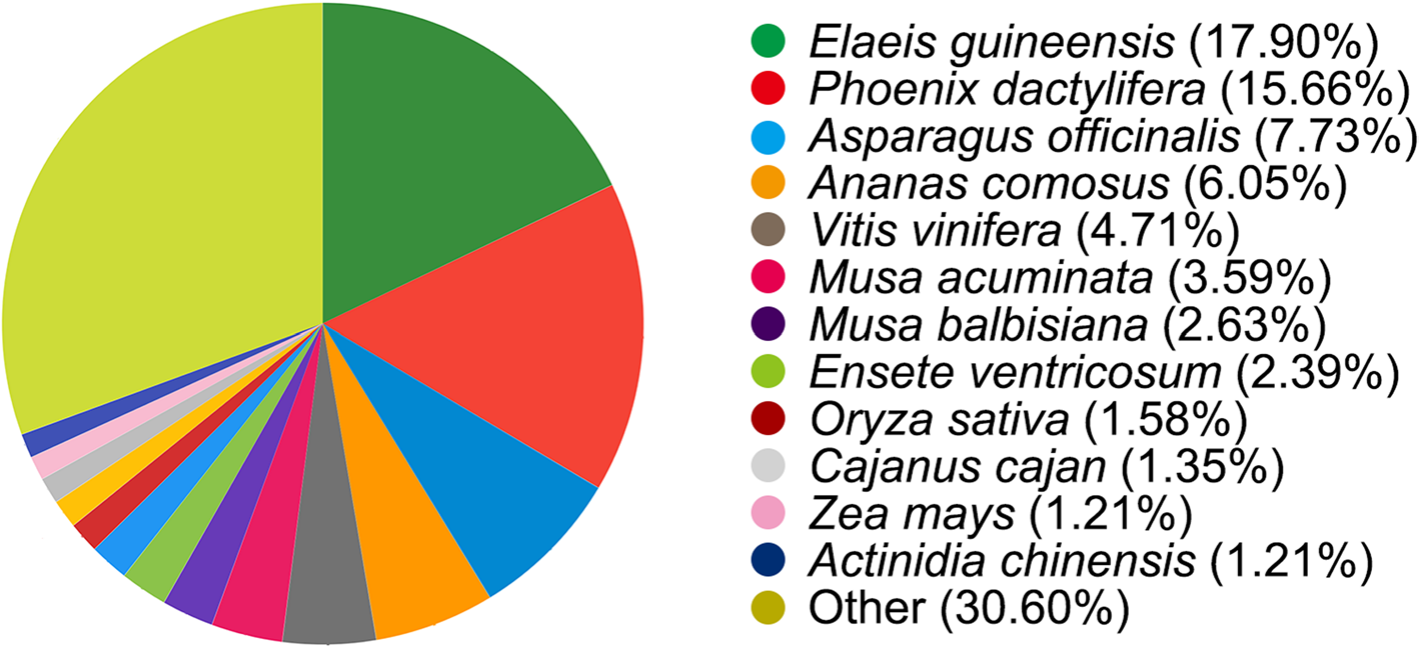
Species distribution of the top BLAST hits by NR annotation for *Lilium longiflorum*‘White Heaven’. The generated transcriptome unigenes were used to blast the NCBI-non-redundant (NR) protein database to match the similar gene sequence of other species. Each sector area represents the similarity between the gene information of lily ‘White Heaven’ and the gene information of other species. The cut-of values for BLAST search was set at 1.0e^− 10^. The cutoff is a threshold of selection criteria to determine whether the genes between species was similar

Based on the NCBI NR database, approximately 31,791 unigenes (57.76%) were successfully annotated. Among the matched plant species, 17.90% of the sequences had top hits to sequences from *Elaeis guineensis*, followed by *Phoenix dactylifera* (15.66%), *Asparagus officinalis* (7.73%), and *Ananas comosus* (6.05%) (Fig. 1).

The heat map of correlation among biological replicates for each treatment (Supplementary Fig. 5 and Supplementary Additional file 1) showed that the biological reproducibility was good. The results of the principal component analysis (PCA) analysis (Supplementary Fig. 6) also showed that there were significant differences among transcriptome profiles of lily leaves subjected to different heat stress treatments. However, there were almost no differences between the 0 h and 0.5 h treatment groups.

**Fig. 2:**
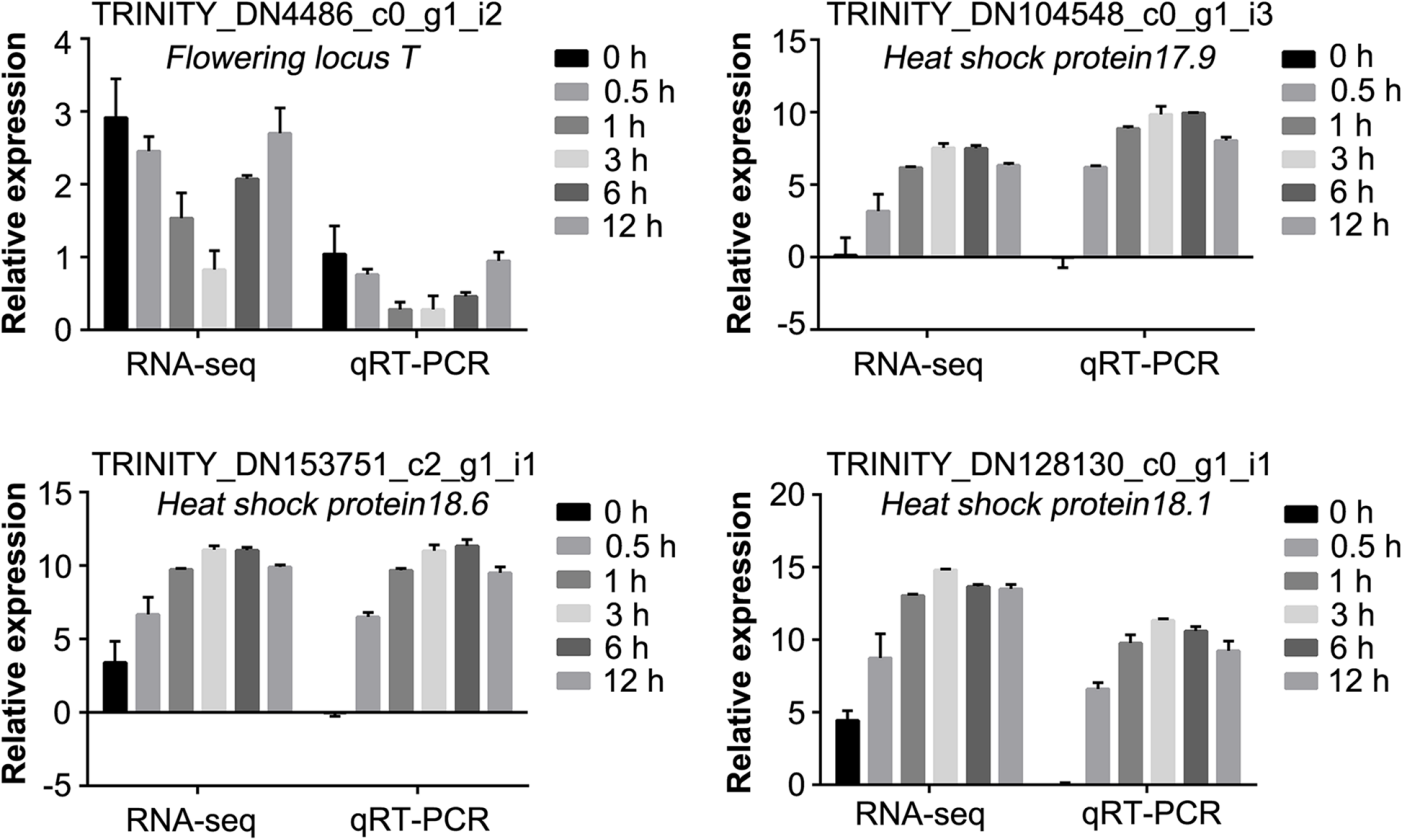
Validation of DEGs by qRT-PCR. The lily housekeeping gene, *18S rRNA* gene, was used as an internal control for normalization in qRT-PCR and relative gene expression levels were calculated using the 2^−ΔΔCT^ method. Data are means ± SD of three biological replicates

Four unigenes were selected for qRT-PCR to validate the accuracy of the RNA-Seq data (Fig. 2). The fold-change in gene expression ratios of these four unigenes between qRT-PCR and RNA-Seq data were significantly positively correlated, indicating that the expression profiles of qRT-PCR were strongly consistent with those obtained from RNA-Seq, suggesting that the data from the transcriptome was reliable.

### DEGs analysis between different pairwise comparison

**Fig. 3:**
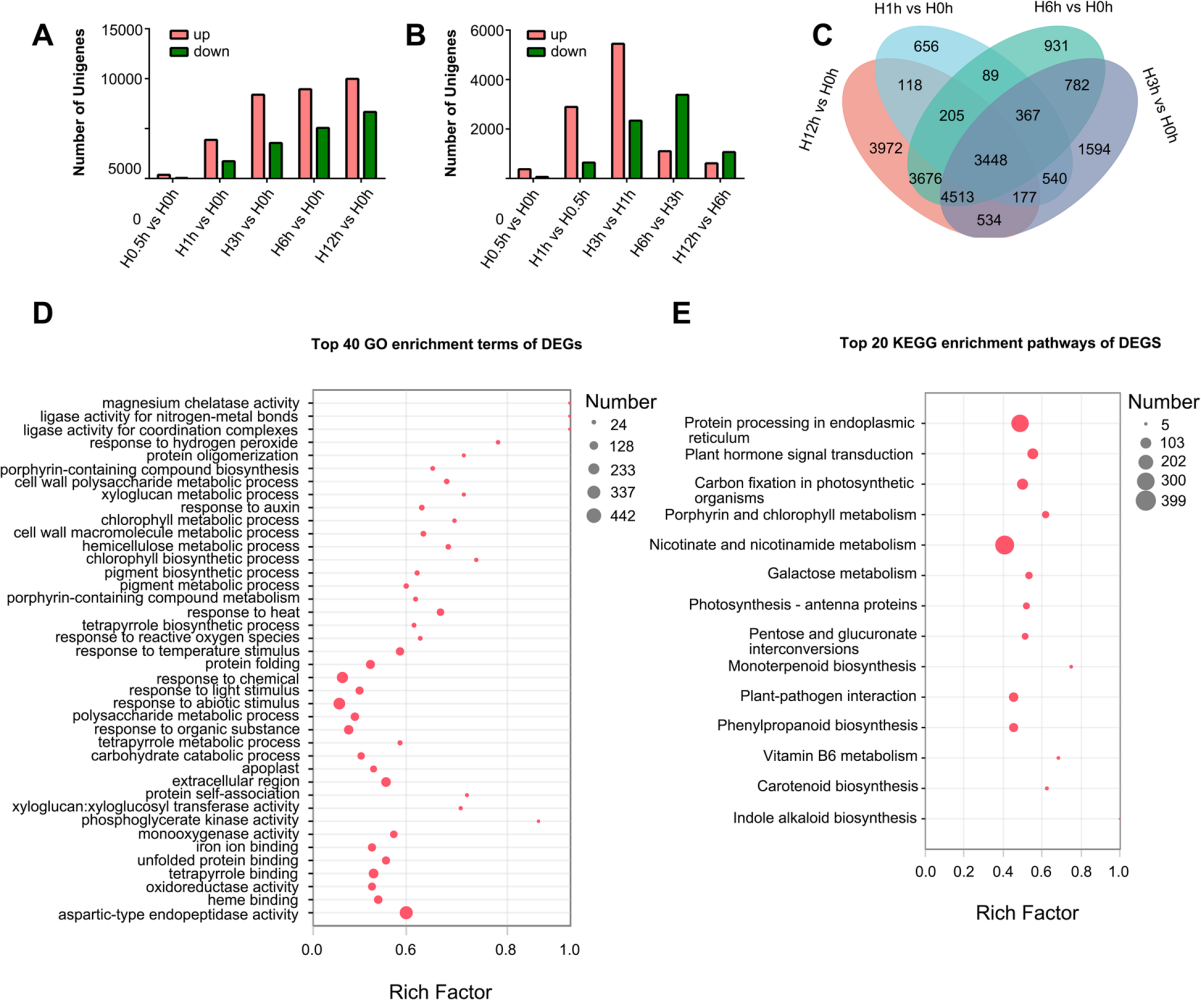
Changes in DEGs numbers in different heat treatments with the GO enrichment and KEGG enrichment analysis cited from Kanehisa M and Goto S (2000) [22]. **A** Changes in DEGs numbers in different pairwise comparison using 0 h group as control. **B** Changes in DEGs numbers in different pairwise comparison between adjacent heat treatments. **C** Overlapping DEGs by Venn analysis among four different pairwise comparison groups with 0 h group as control. **D** 40 GO enrichment terms of DEGs in the GO enrichment analysis combining all the DEGs presented in (**C**) and the unigenes in pairwise comparison groups of 0.5 h vs 0 h. **E** 20 KEGG Enrichment terms of DEGs in the KEGG enrichment analysis combining all the DEGs presented in (**C**) and the unigenes in pairwise comparison groups of 0.5 h vs 0 h. Note: the unigenes in pairwise comparison group of 0.5 h vs 0 h were not analyzed in (**C**) because it displayed few DEGs. In order to identify the most significantly enriched top 40 GO items and top 20 KEGG pathways, the *p* value (FDR value) < 0.001 were selected as the criteria in this analysis. The *p* value represents whether the enriched results are statistically significant and the smaller the *p* value, the more statistically significant it is

Differentially expressed genes (DEGs) among libraries were determined by two sets of pairwise comparisons, one pairwise comparison was using 0 h group as control (0.5 h vs 0 h, 1 h vs 0 h, 3 h vs 0 h, 6 h vs 0 h, and 12 h vs 0 h) and the other was conducted between two adjacent treatments (1 h vs 0.5 h, 3 h vs 1 h, 6 h vs 3 h, and 12 h vs 6 h). The number of DEGs gradually increased to a peak at 12 h (Fig. 3A), while the number of DEGs at 0.5 h vs 0 h (only 432) and 12 h vs 6 h (1681) were fewer than that at 1 h vs 0.5 h, 3 h vs 1 h, and 6 h vs 3 h. The number of DEGs in pairwise comparison of 3 h vs 1 h was the largest in adjacent treatment comparisons (Fig. 3B), which indicated that heat stress had the most significant effect on lily gene expression from 1 h to 3 h at 37 °C. Overlapping DEGs were analyzed using Venn analysis across four adjacent treatment comparisons using 0 h as the control, named as H1 h vs H0 h, H3 h vs H0 h, H6 h vs H0 h, and H12 h vs H0 h; DEGs of 0 h vs 0.5 h were not analyzed because it displayed few DEGs. A total of 3448 common unigenes was identified in all four pairwise comparisons (Fig. 3C).

### GO and KEGG enrichment analysis of DEGs

GO enrichment analysis was used to classify the potential functions enriched among the DEGs (Supplementary Additional file 2). DEGs were classified into GO function enrichment. Among these terms, the high enrichment include: hydrogen peroxide, response to heat, response to reactive oxygen species, response to temperature stimulus, protein folding, abiotic stimuli, apoplast and extracellular region, protein self-association, xyloglucan: xyloglucosyl transferase activity, iron ion binding, and unfolded protein binding (Fig. 3D). KEGG analysis revealed that a total of 1354 DEGs was significantly (*p* ≤ 0.05) enriched in 20 KEGG pathways (Fig. 3E). Genes involved in protein processing in the endoplasmic reticulum formed the largest KEGG enrichment group (340); they could play roles in protein folding, sorting, and degradation. Plant hormone signal transduction (131), carbon fixation in photosynthetic organisms (137), and plant-pathogen interactions (100) were also enriched among these DEGs (Fig. 3E).

**Fig. 4:**
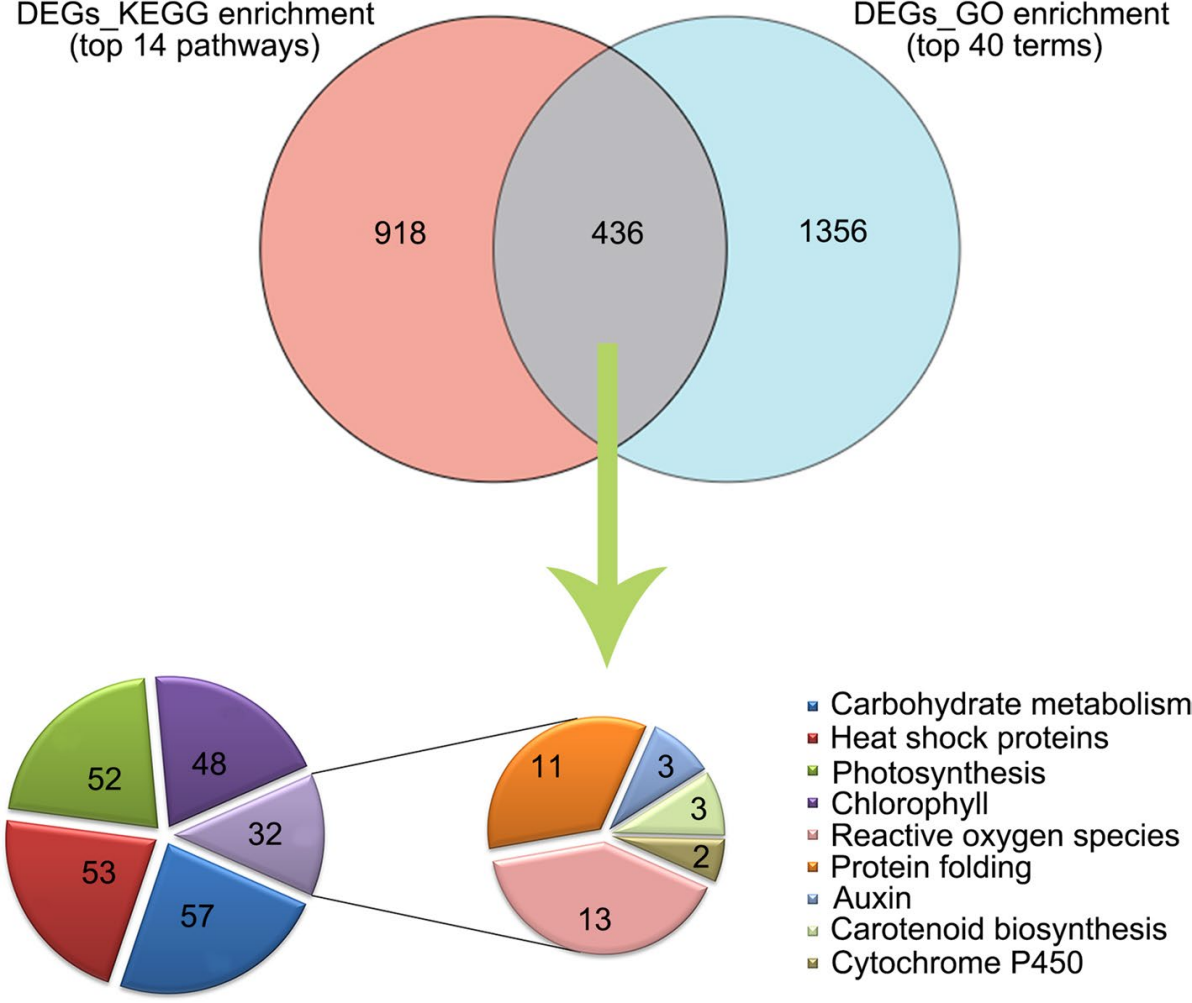
Venn diagram of genes involving in top 40 GO terms with the 14 KEGG pathways. Function distributions of common genes whose expression level were over 2-fold-change at each of heat treatment. The diagram only showed top 14 KEGG pathways under the selection criteria of *p* value (FDR value) < 0.001. The numbers in the sector region represent the number of DEGs involving in corresponding functional items

Combining the genes enriched in of the top 40 GO terms with the 14 KEGG pathways, we identified 436 common genes (Fig. 4). Among these 436 DEGs, the expression level of 242 genes changed over two-fold change under each heat treatment (Supplementary Additional file 3). These 242 genes included 57 genes involved in carbohydrate metabolism and 53 HSP family members (15 *sHSPs*, 12 *HSP70s*, and 26 *HSP90s*) whose expressions were significantly up-regulated. Fifty-two genes were involved in photosynthesis and 48 chlorophyll metabolism-related genes whose expression significantly down-regulated. In addition, 32 genes, including 13 genes associated with ROS (mainly peroxidase), 11 genes involved in the protein folding process, and 3 genes encoding auxin response protein were also significantly regulated.

### Pathways respond to heat stress in lily

**Fig. 5:**
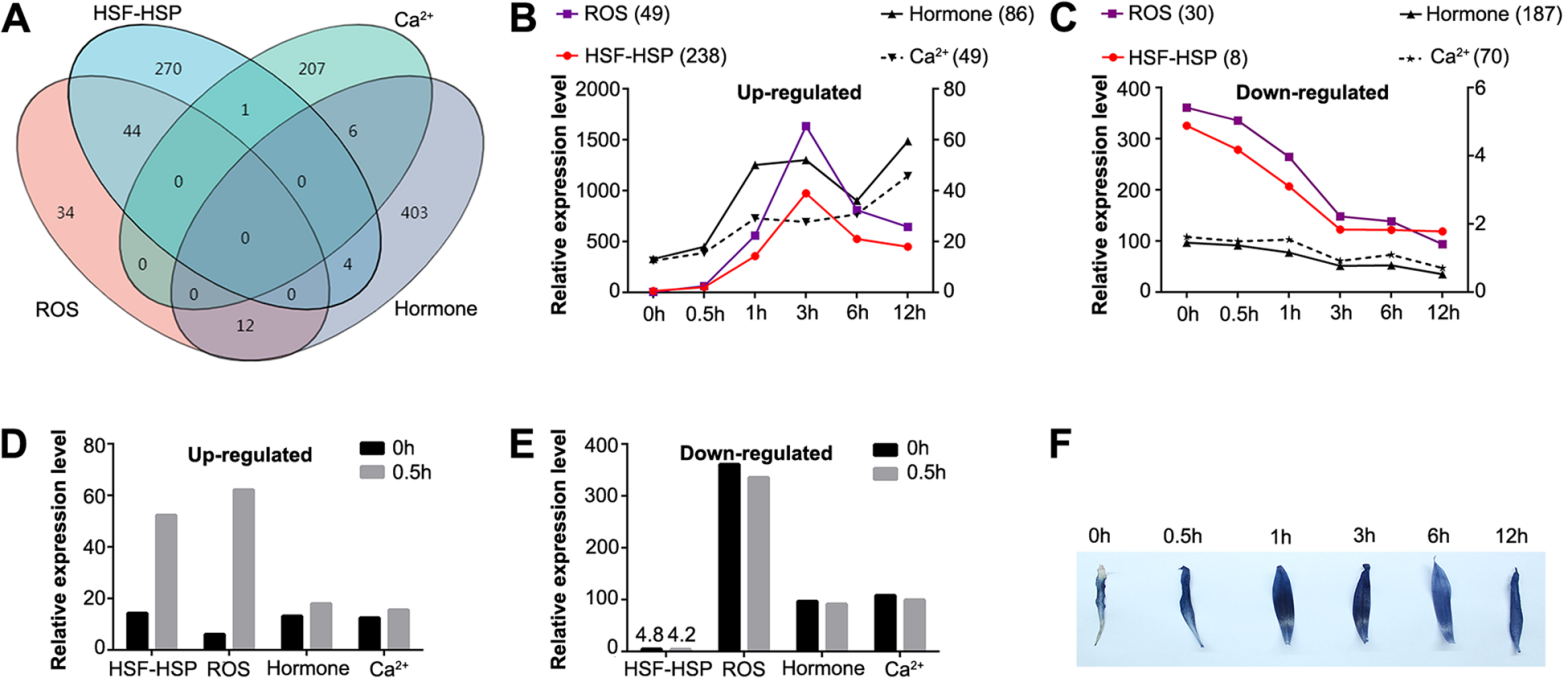
DEGs analysis of four main pathways responded to heat stress. **A** Venn diagram of DEGs of four main pathways. **B** The expression pattern of up-regulated DEGs of four main pathways. **C** The expression pattern of down-regulated DEGs of four main pathways. **D** The expression pattern of up-regulated DEGs of 0 h and 0.5 h of four main pathways. **E** The expression pattern of down-regulated DEGs of 0 h and 0.5 h of four main pathways. **F** The content of O^2−^ in lily leaves of different heat treatments was detected via 0.1%nitro blue tetrazolium (NBT) staining. Note: numbers in brackets of (**B**), (**C**), (**D**) and (**E**) represents the number of DEGs of corresponding pathways. The relative expression level here was calculated by quantitative software RSEM with the quantitative index TPM (transcripts per million reads). In this analysis, the relative expression level was the average fold change for all DEGs of a related group

There are four molecular regulatory mechanisms in plants responding to heat stress: the HSF-HSP, Ca^2+^-CAM, ROS and hormone pathways. The DEGs associated with these four pathways were counted, and Venn analysis was performed. The numbers of DEGs in the four pathways were 319, 214, 90, and 425, respectively. There were no common genes among the four pathways (Fig. 5A). However, the HSF-HSP and ROS pathways had most common genes (44 DEGs) through pairwise comparisons, which suggested that these two pathways were more closely related in response to heat stress in lily. The Ca^2+^-CAM and hormone pathways showed very independent response patterns of heat stress with no shared common genes with other pathways.

The expression patterns of genes in these four pathways were analyzed to reveal their relationship in heat stress response. Up-regulated expressions of DEGs in HSF-HSP and ROS pathway reached a peak at 3 h after heat stress and were significantly higher than that of Ca^2+^-CAM and hormone pathways (Fig. 5B). Down-regulated expressions of DEGs in HSF-HSP and ROS pathway decreased quickly, while the expressions of that in hormone and Ca^2+^ pathways were slow (Fig. 5C). These results show that the expression changes of DEGs were similar between HSF-HSP and ROS pathways, indicating that there were correlations in the HSF-HSP and ROS pathways during heat stress response. The relative expression levels of the DEGs of HSF-HSP, ROS, hormone, and Ca^2+^-CAM pathways at 0.5 h of heat stress was 3.66, 10.18, 1.37, and 1.24 times as high as that at 0 h respectively, indicating that the HSF-HSP pathway and the ROS pathway were very drastic and rapid in response to heat (Fig. 5D, E). Notably, the DEGs of all four pathways could be rapidly induced during 0.5 h–1 h of heat stress. In contrast to the expression patterns of the HSF-HSP and ROS pathways whose up-regulated expression gradually decreased after reaching an induced maximum at 3 h, the expression of DEGs in Ca^2+^-CAM pathway and the hormone pathway gradually up-regulated after 6 h of heat stress. These results showed that the ROS and HSF-HSP pathways might mainly regulate the heat response network in the early stage of heat stress in lily (Fig. 5D). We also detected the ROS level under heat stress using nitro blue tetrazolium (NBT) staining and found that ROS level was increased with the treatment time, which were consistent with the up-regulated expression patterns of DEGs in ROS pathway (Fig. 5F). The ROS level was gradually increased along with the heat treatment times till the 3 h of heat stress and decreased after that, which might be related to the excess accumulation of ROS triggering the removal of ROS. This result indicated that ROS and HSF-HSP pathways might play important roles in the early stage of heat stress response in lilies.

### LlHsfA and LlHsfB family genes were strongly induced by heat stress in lily

**Fig. 6:**
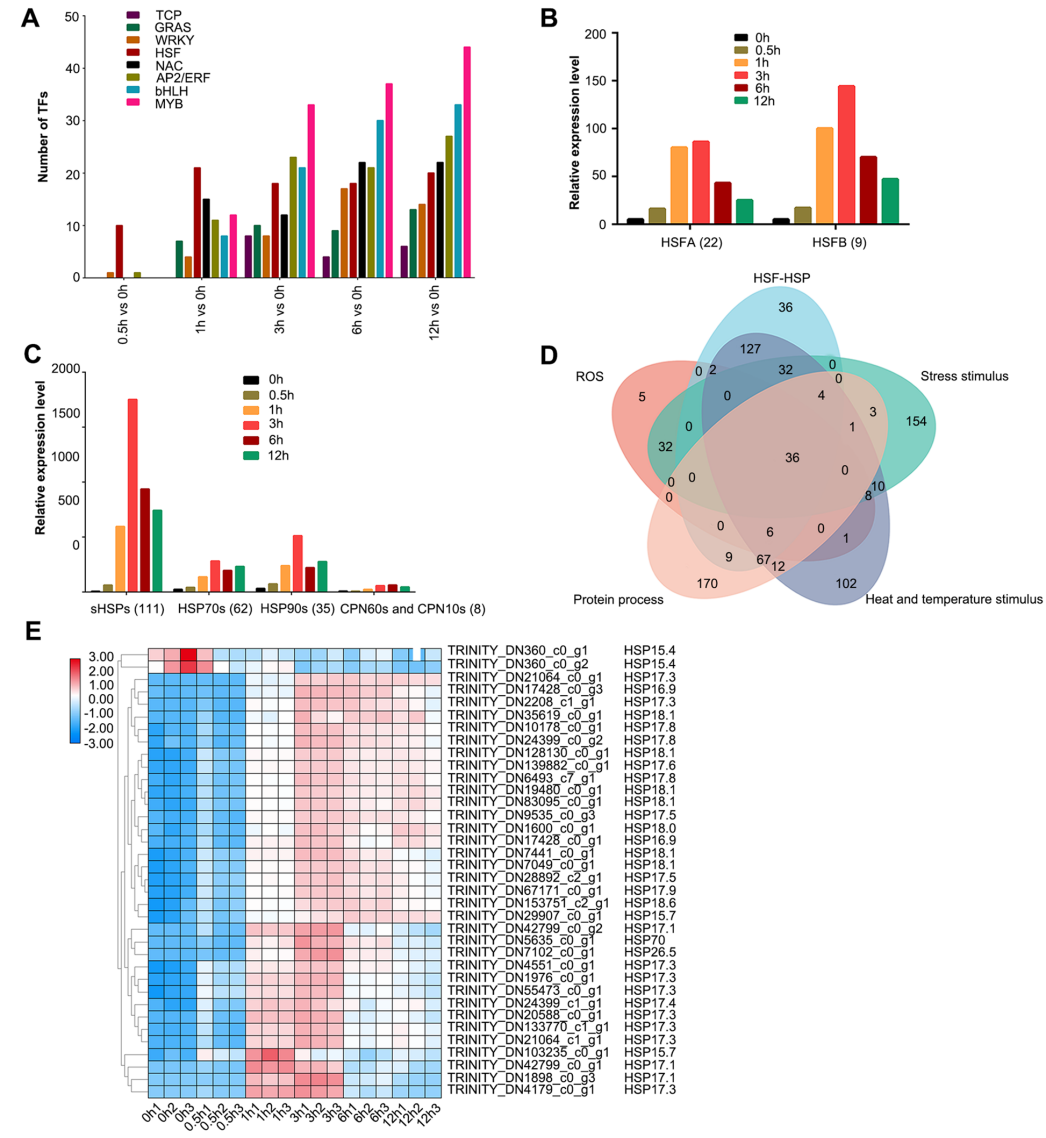
sHSP proteins involved in HSF-HSP and ROS pathway. **A** The response pattern of main differentially expressed transcription factors (TFs) families at different heat stress times. **B** The expression pattern of DEGs of HSF families in lily at different heat treatment times. **C** The expression pattern of DEGs of HSP families in lily at different heat treatment times. **D** Venn diagram of DEGs of main five heat response processes including HSF-HSP pathway process, protein processing, response directly to heat stress process, the response to oxidative stress process, and the ROS pathway process. **E** The heat map of expression pattern of 36 common DEGs of main five heat response processes in **D**. Note: numbers in brackets of (**B**) and (**C**) represents the number of DEGs of corresponding gene family. The relative expression level here was the average fold change for DEGs of a related gene family group

We counted differentially expressed transcription factors (TFs) genes and found that the DEGs of heat stress transcription factor (HSF) family were the dominant transcription factor family genes that responded strongly at 0.5 h after the onset of heat stress (Fig. 6A). After 1 h of heat stress, the DEGs of apetala2/ethylene response factors (AP2/ERF), NAM, ATAF, and CUC transcription factors (NAC), basic helix-loop-helix transcription factors (bHLH), GAI, RGA and SCR transcription factors (GRAS), and v-myb avian myeloblastosis viral oncogene homolog (MYB) families responded strongly to heat stress.

We focused on analyzing the expression patterns of the genes of the HSF family and its target HSP family under heat stress. Of the 319 significantly differentially expressed genes in the HSF-HSP pathway, 246 DEGs were selected for further analysis based on the criteria of *P*-adjust ≤0.05, and a minimal two-fold change in expression level (|log2 Ratio| ≥ 1) at each heat treatment. These 246 DEGs consisted of 31 HSF family genes (12.60%) and 215 HSP family genes (87.40%), including 111 small HSPs family genes (51.63% of total HSP genes), 61 HSP70s family genes (28.37%), 35 HSP90s family genes (16.28%), 6 CPN60 chaperon family (*HSPD1s*), and 2 CPN10 chaperon family (*HSPE1s*) genes (3.72%). In the HSF family, there were 22 HsfA family genes (including 17 A2 family genes, 4 A3 family genes, and 1 A6 family gene), 9 Hsf B family genes (including 4 *HsfB2c*, 4 *HsfB2b*, and 1 *Hsf B2a*) (Fig. 6B). Hsf A and Hsf B family genes were all significantly up regulated at 0.5 h of heat stress. Thus, for the HSF family genes, there were mainly HsfA and HsfB family genes that were significantly induced under heat stress.

### The small heat shock proteins were involved in the crosstalk between HSF-HSP and ROS pathway

Most of the HSP family genes were induced later than the HSF family genes, except for the small heat shock proteins **(**small HSPs**)** family genes, which was consistent with the pattern of HSP regulated by HSFs under heat stress (Fig. 6C). The relative basal expression of small HSPs family genes at 0 h was the lowest (5.72) compared to the HSP70s (24.35), HSP90s (32.24), and CPN60s families (7.85). However, its response to heat stress was significantly more dramatic and earlier than that of other HSP family genes. The expression of small HSPs genes at 0.5 h was 10.87-fold compared to that at 0 h, while the expression of HSP70s, HSP90s, and chaperon family genes at 0.5 h was 1.69, 2.28, and 0.91-fold, respectively, to that at 0 h, indicating that at the onset of heat stress, small HSPs family genes were activated quickly and significantly up regulated.

Moreover, our analysis of 50 most highly induced and repressed genes at each heat treat point suggested that most of the 50 most highly induced genes at each treat point were small HSPs family genes (Supplementary Fig. 7). 32% of DEGs are *small HSPs* genes in 50 most highly induced genes at the heat stress treatment of 0.5 h, following by 56% at 1 h, 67% at 3 h, 38% at 6 h and 26% at 12 h. However, by analyzing the 50 most highly repressed genes at each time point, the DEGs were very different and had rare common pathways at each treat time point. Therefore, these small HSPs were speculated to play a critical role in the HSF-HSP pathway during lilies heat stress response.

Molecular chaperones (like most HSPs) can mitigate the extent of damage to protein structure and stability, as intracellular protein levels, protein stability, and protein structure might be relatively damaged during heat stress. Therefore, a comprehensive Venn analysis were performed about common *HSPs* genes among the DEGs of the HSF-HSP pathway, DEGs related to protein processing (308 DEGs), DEGs that respond directly to heat stress (408 DEGs), DEGs associated with the response to oxidative stress (280 DEGs), and the 90 DEGs of the ROS pathway. From the Venn analysis of these five combinations, 36 HSF-HSP pathway DEGs were found to be involved in all above five heat response processes used for this Venn analysis, including 35 small HSPs family genes and 1 HSP70 family gene (Fig. 6D, E), which reveals the dominant role of the small HSPs family in lily heat response.

### Decreased expression of LlHsfA2 caused thermal sensitivity and high level of ROS-scavenging enzymes in lily

**Fig. 7:**
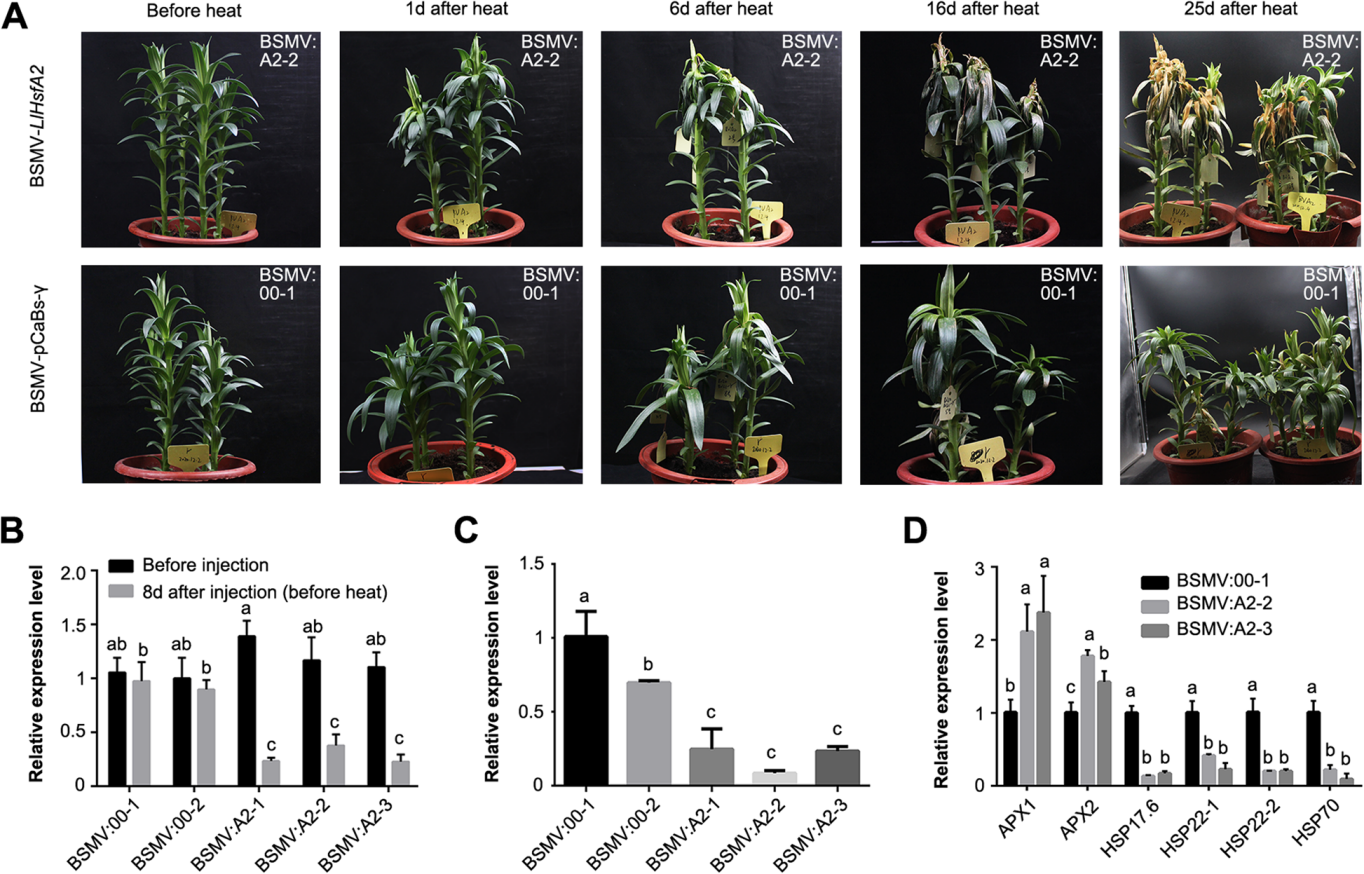
Decreased expression of *LlHsfA2* caused sensitive phenotype of heat stress. **A** Thermotolerance of *LlHsfA2*-silenced lily lines was reduced after 24 h heat stress of 42 °C. **B** The detection of mRNA level of *HsfA2* in *LlHsfA2*-silenced lily lines, control lily lines and wild type (before injection) by qRT-PCR. **C** The detection of mRNA level of *LlHsfA2* in the leaves of *LlHsfA2* silenced lily lines and control lily lines after 1d of heat stress by qRT-PCR. **D** The detection of mRNA level of *LlHsfA2* putative down-stream genes in *LlHsfA2*-silenced lily lines after 1d of heat stress by qRT-PCR. The lily housekeeping gene, *18S rRNA* gene, was used as an internal control for normalization in qRT-PCR and relative gene expression levels were calculated using the 2^−ΔΔCT^ method. Data are means ± SD of three biological replicates. Different letters indicate significant differences between groups at *P* < 0.05 according to Duncan’s multiple range test

Finally, a potential regulator of small HSPs gene, *LlHsfA2* (TRINITY_DN1223_c0) was selected for the barley stripe mosaic virus-virus inducing gene silence (BSMV -VIGS) experiment to verify its function in response to heat stress in lily, as which was induced sharply by heat (Supplementary Fig. 8) and was the top HSF family among the number of DEGs involved in heat stress. It has previously been reported that *LlHSP17.6* (TRINITY_DN139882_c0) and *LlAPX1* (TRINITY_DN3152_c0), which also responded to ROS signals in the RNA-seq analysis as the common genes of the HSF-HSP and ROS pathways, functioned as the target genes of *LlHsfA2* in transgenic *Arabidopsis thaliana* [23, 24]. The thermotolerance of *LlHsfA2* silenced lily lines was reduced as the leaves turned yellow and died earlier than those of the control plants, with the extension of heat stress treatment duration; while the control plants were still alive (Fig. 7A). Expression of *LlHsfA2* in BSMV silenced lines was significantly down-regulated according qRT-PCR result (Fig. 7B, C), suggesting that the thermal sensitivity of *LlHsfA2* silencing lily was caused by the decreased expression of *LlHsfA2*, which was consistent with the previous results that *LIHsfA2* enhanced the thermotolerance of transgenic *Arabidopsis thaliana* [23].

The expressions of HsfA2 putative down-stream genes, *LlHSP17.6*, *LlHSP22* (TRINITY_DN35718_c2 and TRINITY_DN3965_c0), and *LlHSP70* (TRINITY_ DN12982_c0) were examined in *LlHsfA2* silenced plants using qRT-PCR, and were shown to be significantly down-regulated (Fig. 7D), consistent with previous reports [23]. While the expression of *LlAPX1* and *LlAPX2* (TRINITY_DN11489_c0) were up-regulated in silenced lily lines, which is conflicting with *AtAPX2* being up-regulated in *LlHsfA2* over-expressing transgenic *Arabidopsis thaliana* [23]. It might be related to the different stages of sample and treatment times after heat stress, which indicates there might be different regulation mechanisms between *LlHsfA2* and *APX2* in lilies and transgenic *Arabidopsis* plants based on the species difference.

## Discussion

Heat stress seriously inhibited growth and the flower quality and quantity of lily. Bulbous plant species differ greatly from annuals in their life cycle and physiological requirements, and therefore might have different molecular mechanisms in heat stress response [25, 26]. So far, only limited information is available on the molecular aspects of thermotolerance in geophytes plants like lily. Several genes in lily which is the famous bulbous plant with great ornamental and economic values, such as *LlHsfA2*, *LlHsfA1*, *LlHsfA3*, *LlCaM3*, and *LimHSP16.45*, were cloned and found to regulate different signaling pathways of the heat stress response networks [23, 24, 27–29].

However, with the large genome and different germplasm resources, detailed transcriptome studies about molecular mechanisms of response to heat stress in bulbous plants like lily are scarce. So, it is necessary to explore mechanism of response to heat stress in monocotyledonous geophytes plants which will provide meaningful information for the further study of heat tolerance of lily. Therefore, *Lilium longiflorum* ‘White Heaven’ plants were heat-treated with different times for RNA-seq in this study. A total of 365.89 Gb clean reads were generated and 55,044 unigenes were obtained and annotated, which were close to the previously reported data of *L*. *orential* ‘Siberia,’ Asiatic hybrid lily cultivar ‘Tiny Padhye’ and *L*. *distichum* [18, 30–32]. 3448 DEGs were found in different pairwise comparisons four unigenes from which were significantly positively correlated between qRT-PCR and RNA-Seq (Fig. 2). These results suggested that heat stimuli significantly affected the transcriptome profiles in lily and could provide more meaningful information for the further study of heat tolerance in bulbous plants.

GO enrichment analysis reveals that DEGs were involved in response to heat, reactive oxygen species, temperature stimulus, protein folding, and abiotic stimuli, which were the top enriched function terms (Fig. 3D). KEGG analysis reveals that 1354 DEGs were significantly enriched in protein processing in the endoplasmic reticulum, plant hormone signal transduction, carbon fixation in photosynthetic organisms, and so on (Fig. 3E). In the GO and KEGG analyses, 436 shared common genes were found, which were related to carbohydrate metabolism, HSP, photosynthesis, ROS, and protein folding (Fig. 4). These results indicate that signal pathways related to heat stress were multiple and complex in lily, which are the same with model plants such as *Arabidopsis* and rice [6, 7, 33]. However, these results were important for bulb plants as providing reference resources for molecular research in the future.

### Expression pattern of DEGs in HSF-HSP pathway is identical in heat stress response in lily

Previous studies suggested that plants firstly perceive the high temperature signal through the plasma membrane, which promotes a large influx of Ca^2+^ to form the primary signal [6]. ROS played an important role in the amplification of the primary heat signaling cascade, which ultimately transmitted the heat signal to the nucleus to activate transcriptional regulatory networks such as HSFs to regulate downstream genes encoding HSFs, HSPs, ROS scavenging enzymes, metabolic enzymes, and hormone production [13].

In the present study, the relative expression of HSF-HSP, ROS, hormone, and Ca^2+^-CAM pathways at 0.5 h of heat stress was 3.66, 10.18, 1.37, and 1.24 times as high as that at 0 h respectively, indicating that the HSF-HSP pathway and the ROS pathway responded drastically and rapidly to heat (Fig. 5D). Previous studies showed that *HsfA1* could be induced in 10 min of heat stress treatment in *Arabidopsis* and 5 min after the onset of heat treatment in wheat [34, 35], which was consistent with the phenomenon of HSFs in our study (Fig. 5D). Rapid and systemic whole-plant transmission of electric, calcium, and ROS waves was recently shown to be required for plant acclimation to heat or light stresses, as well as for systemic wound responses [36–39]. The results of the present study also present the phenomenon of rapid induction of ROS signals by heat stimuli (Fig. 5D, F), consistent with the reported study by Zandalinas [33]. Moreover, up-regulated expressions of HSF-HSP and ROS pathway-related genes both peaked at 3 h after heat stress and were significantly higher than that of the Ca^2+^-CAM and hormone pathways (Fig. 5B). Down-regulated expression of HSF-HSP and ROS pathway-related genes decreased quickly, while the expression of hormone and Ca^2+^ pathways genes decreased slowly (Fig. 5C). All these results indicate that genes in HSF-HSP and ROS responded quickly to heat stress and were dominated thermal response pathways in lily. Interestingly, the results of the present study not only presented the phenomenon of rapid induction of genes in HSF-HSP pathway and ROS pathway by heat stimuli but also their similar expression pattern in response to heat stress with the highest number of common genes (44 DEGs) (Fig. 5A-E). These results mean there might be certain crosstalk between these two pathways in lily.

### The sHSPs family genes were earlier induced than other HSPs family genes in response to heat in lily

In our study, the most significantly enriched pathways and GO terms in DEGs after heat stress in lily were both intracellular protein-related processes, such as protein folding and protein binding. HSPs act as molecular chaperones, reducing the aggregation and resolubilization of denatured proteins, promoting the folding of nascent polypeptides, and facilitating the refolding of denatured proteins [40].

Existing studies have shown that HSP90s and HSP70s families play very important roles during heat stress. However, only few researches have been conducted on how the small heat shock proteins (small HSPs or HSP20s) family functions during heat stress in plants [41]. Plants synthesize a class of low molecular weight heat shock proteins (15–30 kD), referred to as small heat shock proteins (small HSPs), in response to adversity and developmental signals [42]. In general, plant small HSPs have a molecular chaperone function, which prevents the accumulation of denatured proteins in the absence of ATP, thus playing an important role in plant resistance and adaptation to stressful environments.

In our analysis, the small HSPs family was involved in important molecular processes in the rapid response to lily heat stress (Fig. 6C). Although the small HSPs family genes had the lowest relative expression at 0 h among the HSP family genes, its response to heat stress was significantly more dramatic and earlier than that of other HSPs family genes. After 0.5 h of heat stress, the relative expression of small HSPs family genes reached 10.87-fold of that of 0 h, while the relative expression of HSP70s and *HSP90s* at 0.5 h was about 2-fold, indicating that the small HSPs family genes responded positively to heat stress at the onset of heat stress and significantly up-regulated the gene expression (Fig. 6C). These results were consistent with those of Nollen and Morimoto [43], who found that the DEGs involved in protein processing in the endoplasmic reticulum were significantly enriched after 5 min HS treatment in leaves and 10 min in grain, strongly supporting the hypothesis that the HSPs competition model plays a primary role in early HS sensing and signaling. Our results indicate that small HSPs family genes play primary roles in early heat stress sensing and signaling.

### The small HSPs family genes were involved in the crosstalk between HSF-HSP and ROS pathway in lily

Interestingly, our analysis also revealed that small HSPs genes were not only involved in important molecular processes but also in the ROS pathway in the rapid response to heat stress in lily (Fig. 6D). In the Venn analysis of DEGs (Fig. 6D, E), there were 35 small HSPs family genes involved in ROS pathway. As a small HSPs family gene, *LimHSP16.45* could protect plants against high temperatures by scavenging cellular ROS in David Lily (L. *davidii Raffill* var. Willmottiae) [27]. In addition, *PfHSP21.4* and *CaHSP16.4* could enhance heat tolerance by increasing the activity of superoxide dismutase and peroxidase in primula and pepper (*Capsicum annuum* L.) [44, 45]. These small HSPs family genes were all involved in heat stress via the ROS scavenging pathway, which was consistent with the results of small HSPs family genes in RNA seq using *L. longiflorum*. Therefore, it was possible that these small HSPs family genes play also critical roles in the crosstalk between the HSF-HSP and ROS pathways.

In the HSF-HSP pathway, one upstream transcription factor of small HSPs family genes is HsfA2, which has been reported to be involved in the heat stress response in *Arabidopsis*, tomato, lily and so on [23, 46, 47]. Also, some studies have found that HsfA2 can alleviate oxidative damage caused by heat stress by regulating ROS scavengers, such as *Ascorbate Peroxidase* (*APX*) for example [23, 48–51]. So, we speculated that small HSPs family genes might contribute to the crosstalk between the HSF-HSP and ROS pathways through *LlHsfA2* mediated molecular regulation modules in lily. The *LlHsfA2* silenced lines by BSMV-VIGS were sensitive to heat stress (Fig. 7A), which was consistent with the results of evaluated thermotolerance in *Arabidopsis* by over-expression of *LlHsfA2* [23]. And *LlHsfA2* silenced lines not only reduced expression of *LlHsfA2* downstream targets such as *LlHSP70*, *LlHSP22.1*, *LlHSP22.2*, and *LlHSP17.6* (Fig. 7D) but also directly elevated the expression of the ROS scavenger *LlAPX1* and *LlAPX2*. The expression of *LlAPX1* and *LlAPX2* was inconsistent with previous studies that the expression of *AtAPX2* was significantly induced in the *LlHsfA2* transgenic Arabidopsis plants [21]. This conflict could be caused by different heat stress condition and sampling time, or upstream transcription factors of *APX* could be more than one HsfA2, Anyway, these all need more experiment to identify. This indicates that the BSMV-VIGS system can be used to identify gene function and *LlHsfA2* is involved in the heat stress response through both HSF-HSP pathway and ROS pathway, which may play an important role in small HSPs-mediated crosstalk in lily. Future studies will focus on the molecular mechanism of regulating the expression of small HSPs family genes by LlHsfA2.

**Fig. 8:**
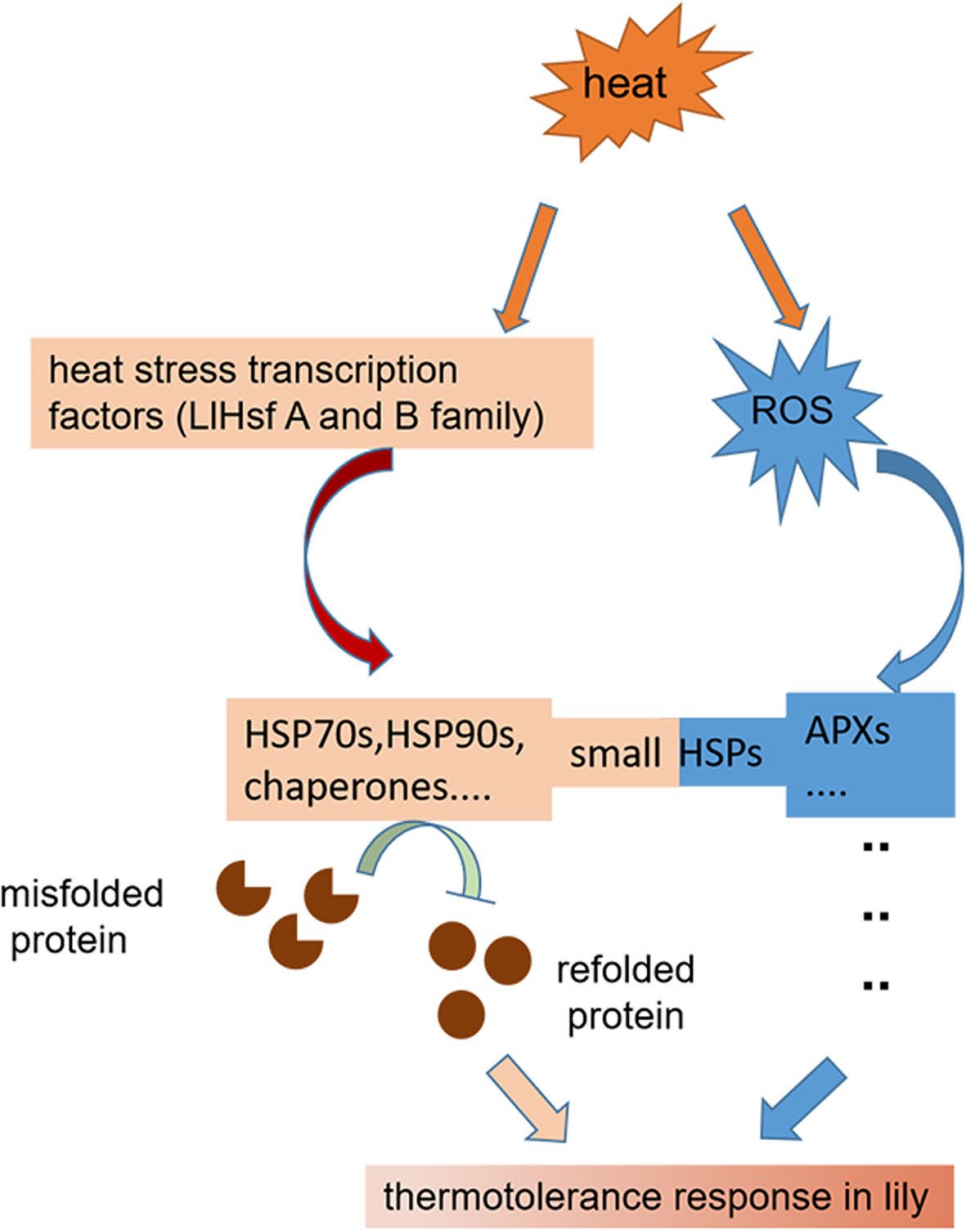
Proposed model of the HSF-HSP pathway and ROS pathway involving in response to heat stress in lily. Heat stress induced the expression of numerous DEGs including the genes of HSF-HSP pathway and ROS pathway which responded quickly and strongly to the heat stress in the transcriptome expression profile. Moreover, these two pathways shared some common DEGs belonging to sHSPs family during heat stress response indicating that sHSPs family play an important role in regulation mechanisms of heat stress response in lily

At last, based on our transcriptomic analysis, a model was proposed about heat stress response in lily (Fig. 8): the heat stress induced heat stress transcription factors such as HsfA family to regulate expression of HSP genes. At the same time, ROS increased under heat stress, and small HSPs were found to respond in the ROS signaling. Small HSPs were the critical juncture in crosstalk between HSF-HSP and ROS pathways during heat stress response in lily. While the function of small HSPs in ROS signaling needs research deeply in future.

## Conclusion

We provided transcriptomic data on the response of lily to heat stress with different treat time in bulbous plants. A total of 365.89 Gb clean reads was generated and 55,044 unigenes were obtained and annotated, which will provide reference resources for gene isolation in heat stress research in lily. More importantly, the HSF-HSP pathway and ROS pathway play important roles and have crosstalk in which sHSPs family genes were the critical juncture during heat stress response. Future studies to confirm the molecular function of these genes will provide new data for understanding the regulation mechanism of HSR in lily and other plants.

## Methods

### Plant materials

Based on previous studies [24], the expression of heat response-related genes was more significantly expressed in lily leaves than in roots and bulbs under heat stress. Therefore, we selected lily leaves as samples for the RNA-seq analysis. The aseptically grown seedlings of *L. longiflorum* ‘White Heaven’ cultured for 37 days (with 4–6 leaves and approximately 1 cm bulb diameter), were used in RNA-seq experiments. The plants were placed in an incubator at 37 °C (in a 12 h-light/12 h-dark cycle) for different heat treatments (0 h, 0.5 h, 1 h, 3 h, 6 h, and 12 h). The leaves were collected immediately after heat treatment from three biological replicates and immediately put into liquid nitrogen for flash freeze, stored at − 80 °C for RNA extraction. No permissions were necessary to collect the plants.

### RNA isolation, library preparation, and sequencing

Total RNA was extracted using TRIzol Reagent (Life Technologies, USA) and purified with the RNAprep Pure Plant Plus Kit (Polysaccharides & Polyphenolics-rich, TIANGEN BIOTECH CO., LTD, Beijing, China). RNA completeness and concentration were detected by 1% Agarose gel electrophoresis and a NanoDrop 2000 spectrophotometer (Thermo Fisher Scientific, USA). RNA was reverse-transcribed into cDNA. The cDNA libraries were then sequenced on an Illumina Hiseq 2000 platform by Novogene Co., Ltd. (Beijing, China). Library quality was evaluated using the Fastx_toolkit_0.0.14. The quality control of the original sequencing data was conducted using SeqPrep, and high-quality clean data were obtained for subsequent analysis using Sickle. Data assembly and unigene annotation were performed by Majorbio Co., Ltd. (Shanghai, China). Short reads were assembled using Trinity software. Optimized filtering of assembly results was conducted using TransRate and CD-HIT. Assembly quality was evaluated using Benchmarking Universal Single-Copy Orthologs. Unigene sequences were mapped to the assembled sequences using SOAPaligner/soap2.

### Unigene annotation

Gene functions were annotated by searching public databases, including NCBI non-redundant protein (NR), Clusters of Orthologous Groups of proteins (COG), Swiss-Prot, Gene Ontology (GO), Kyoto Encyclopedia of Genes and Genomes (KEGG), and Pfam (protein family). Transcription factors were predicted by searching the plant TFDB using HMMER3. Principal component analysis (PCA) plot construction was performed using the R package. The dataset is available in the NCBI Short Read Archive under the accession number PRJNA699023, which contains 18 RNA-seq data (accession numbers SAMN17764925-SAMN17764942) from 18 libraries.

### Analysis of differentially expressed genes

Gene expression levels were estimated using RNA-Seq by Expectation-Maximization [22]. The number of unigene-matched reads was normalized by calculating the TPM (transcripts per million reads) values in different samples. Comparison of unigene expression among the different samples was performed using the DESeq 2R package Criteria for classification as significant DEGs included P-adjust ≦ 0.05 [52] and unigenes with a minimal two-fold difference in expression (|log_2_ Ratio| ≥ 1). Notably, in this study, the relative expression is the average fold change for all DEGs of a related group or a related gene family.

### GO and KEGG pathway enrichment analysis

For GO and KEGG enrichment analysis, DEGs were mapped to GO terms in the Gene Ontology database using WEGO and KEGG orthology terms (www.kegg.jp/kegg/kegg1.html) in the KEGG pathway database [53, 54] using the BLASTALL software. In this GO and KEGG enrichment analysis of DEGs, all the DEGs generated from the different comparison groups including 0.5 h vs 0 h, 1 h vs 0 h, 3 h vs 0 h, 6 h vs 0 h and 12 h vs 0 h were combined for the analysis. All expressed genes in the transcriptome were annotated based on BLAST search and searched against the Swiss-Prot database. In addition, in order to identify the most significantly enriched top 40 GO items and top 20 KEGG pathways, we selected the corrected *p* value (FDR value) < 0.001 as the criteria in this analysis. The *p* value represents whether the enriched results are statistically significant and the smaller the *p* value, the more statistically significant it is.

### Quantitative real-time PCR verification

To verify the quality of the RNA-Seq data, eight DEGs were selected for qPCR analysis. Lily housekeeping gene, *18S rRNA* gene, was used as an internal control for normalization. Primers for qRT-PCR were designed using Premier 6.0, and are shown in Supplementary Additional file 4. Relative gene expression levels were detected using the SYBR® Green Real-Time PCR Master Mix according to the manufacturer’s instructions on a Step One plus TM Real-Time PCR System. The relative expression level of each gene was calculated using the 2^−ΔΔCT^ method [55]. Three biological replicates and three technical replicates were performed for each sample.

### Detection of relative ROS content and chlorophyll content of lily leaves under heat stress

Leaves of the aseptically grown seedlings of *L. longiflorum* ‘White Heaven’ cultured for 37 days (with 4–6 leaves and approximately 1 cm bulb diameter), were collected for ROS and chlorophyll content detection immediately after different high-temperature treatments (0 h, 0.5 h, 1 h, 3 h, 6 h, and 12 h) at 37 °C (in a 12 h-light/12 h-dark cycle). The O^2−^content in leaf samples was detected via nitro blue tetrazolium (NBT) staining [56, 57]. Chlorophyll content was measured using SPAD-502 Plus (KONICA MINOLTA SENSING INC., JAPAN) according to the manufacturer’s protocol.

### Verifying the function of lily *HsfA2* by BSMV silencing system

The barley stripe mosaic virus (BSMV) vectors (pCaBS-α, pCaBS-β, and pCaBS-γ) under the control of a double 35S promoter, were donated by Prof. Dawei Li, College of Biological Sciences, China Agricultural University [58]. The target fragment with the length of 276 bp of *HsfA2* gene was cloned into the pCaBS-γ LIC (Ligation Independent Cloning) vector and transferred into *Agrobacterium* spp. strain EHA105 purchased from Biomed Gene Technology Co., Ltd. (Beijing, China). Primers used as follows: BSMV: LlHsfA2-forward: AAGGAAGTTTAAGCCAAGGCGGTATTCGAA and BSMV: LlHsfA2-reverse: AACCACCACCACCGTGCGACAAATCAAACGACATGG. Agrobacterium cells contain pCaBS-α, pCaBS-β, pCaBS-γ or LlHsfA2-pCaBS-γ vectors were cultured in liquid LB and suspended using suspension solution (MgCl_2_ 10 mmol•L^− 1^, MES 10 mmol•L^− 1^ and AS 200 μmol•L^− 1^, pH 5.6) to OD_600_ = 1.0, respectively. Then suspension of pCaBS-α, pCaBS-β and pCaBS-γ-LlHsfA2 or pCaBS-γ were mixed according to the ratio of 1:1:1 and placed in the dark environment of 28 °C for 5 h for injecting. Subsequently, *Agrobacterium* was injected into lily leaves to silence *LlHsfA2* expression. Leaves of lily plants were collected for qRT-PCR to verify gene silencing 8–9 days after injection. *LlHsfA2*-silenced plants were subjected to heat treatment at 42 °C for 24 h and then incubated at 22–23 °C for heat stress phenotype observation.

## Supplementary Information

**Additional file 1.** The information of all the annotated 55,044 unigenes in this RNA-seq data.

**Additional file 2.** DEGs for GO and KEGG enrichment analysis.

**Additional file 3. **436 common genes enriched in of the top 40 GO terms and the 20 KEGG pathways.

**Additional file 4.** primers sequence used in this study.

**Additional file 5:** **Figure S1.** Relative chlorophyll content of lily leaves using SPAD-502 Plus after different heat stress (0 h, 0.5 h, 1 h, 3 h, 6 h, and 12 h). **Figure S2.** Unigene functions were annotated by COG (Clusters of Orthologous Groups of proteins) databases. **Figure S3.** Unigene functions were annotated by GO (Gene Ontology) databases. **Figure S4.** Unigene functions were annotated by KEGG (Kyoto Encyclopedia of Genes and Genomes) databases. **Figure S5.** Heat map of correlation among biological replicates for each treatment. **Figure S6.** Principal component analysis (PCA) among biological replicates for each treatment. **Figure S7.** The distribution of small heat shock protein family genes in the top up-regulated 50 DEGs of different heat treatment groups. **Figure S8.** The relative expression levels of *LlHsfA2* in lily leaves exposed to different heat stress treatments (0 h, 0.5 h, 1 h, 3 h, 6 h, 12 h, 24 h and 48 h). **Table S1.**Quality control and assembly assessment of sequencing data. **Table S2.** Evaluation of assembly and distribution of unigene length.

## Data Availability

Most data generated or analyzed during this study are included in this published article and its supplementary information files. The opened RNA-seq data (accession numbers SAMN17764925-SAMN17764942) used and analyzed during this study are available in the NCBI database.

## Abbreviations

AP2/ERF: Apetala2/ethylene response factors
APX: Ascorbate peroxidase
bHLH: Basic helix-loop-helix transcription factors
BSMV: Barley stripe mosaic virus
COG: Clusters of Orthologous Groups of proteins
DEGs: Differentially expressed genes
GO: Gene Ontology
GRAS: GAI, RGA and SCR transcription factors
HSF: Heat stress transcription factor
HSP: Heat shock proteins
HSR: Heat stress response
KEGG: Kyoto Encyclopedia of Genes and Genomes
MYB: V-myb avian myeloblastosis viral oncogene homolog
NAC: NAM, ATAF, and CUC transcription factors
NR: Non-redundant protein
Pfam: Protein family
PCA: Principal component analysis
ROS: Reactive oxygen species
