# Supplementary material for: Small HSPs play an important role in crosstalk between HSF-HSP and ROS pathways in heat stress response through transcriptomic analysis in lilies (Lilium longiflorum)
Source: BMC Plant Biol. 2022 Apr 19;22:202. doi: 10.1186/s12870-022-03587-9 (PMC9017035; doi:10.1186/s12870-022-03587-9)
Supplement: Supplementary file 5 — Additional file 5: Figure S1. Relative chlorophyll content of lily leaves using SPAD-502 Plus after different heat stress (0 h, 0.5 h, 1 h, 3 h, 6 h, and 12 h). Figure S2. Unigene functions were annotated by COG (Clusters of Orthologous Groups of proteins) databases. Figure S3. Unigene functions were annotated by GO (Gene Ontology) databases. Figure S4. Unigene functions were annotated by KEGG (Kyoto Encyclopedia of Genes and Genomes) databases. Figure S5. Heat map of correlation among biological replicates for each treatment. Figure S6. Principal component analysis (PCA) among biological replicates for each treatment. Figure S7. The distribution of small heat shock protein family genes in the top up-regulated 50 DEGs of different heat treatment groups. Figure S8. The relative expression levels of LlHsfA2 in lily leaves exposed to different heat stress treatments (0 h, 0.5 h, 1 h, 3 h, 6 h, 12 h, 24 h and 48 h). Table S1.Quality control and assembly assessment of sequencing data. Table S2. Evaluation of assembly and distribution of unigene length. [file 12870_2022_3587_MOESM5_ESM.doc]

**Supplementary Material**

**Supplementary Figures**

**
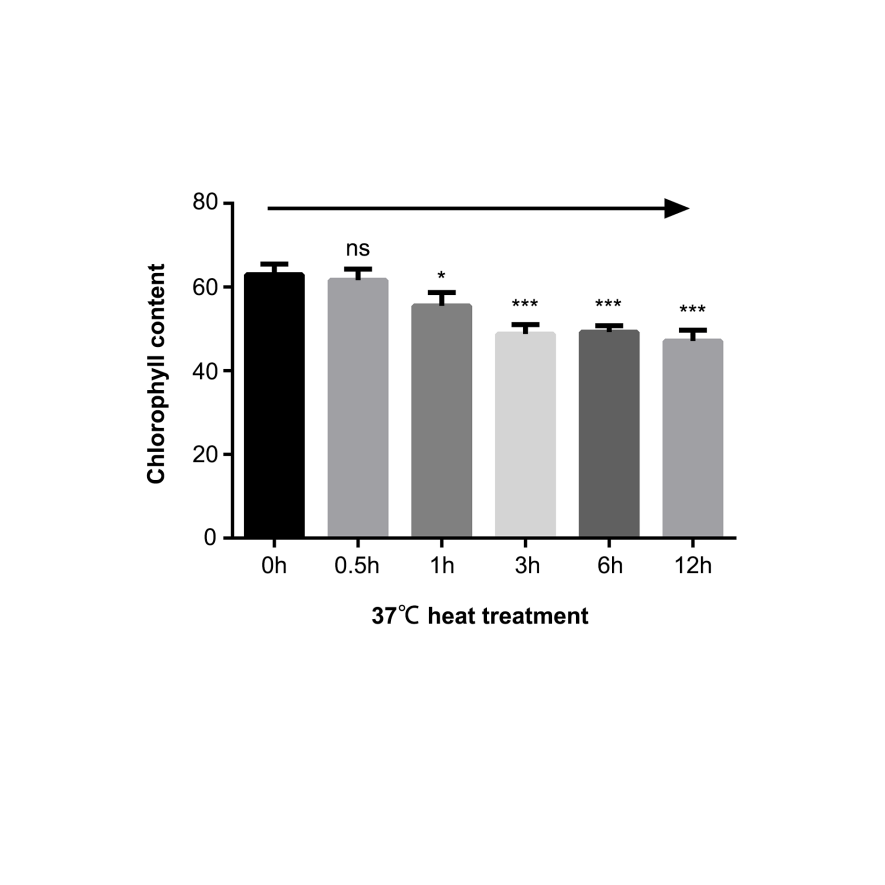
**

**Supplementary Figure 1.**Relative chlorophyll content of lily leaves using SPAD-502 Plus after different heat stress (0 h, 0.5 h, 1 h, 3 h, 6 h, and 12 h). The aseptically grown seedlings of *L*. *longiflorum* ‘White Heaven’ cultured for 37 days (with 4-6 leaves and approximately 1 cm bulb diameter) were used as the materials here.


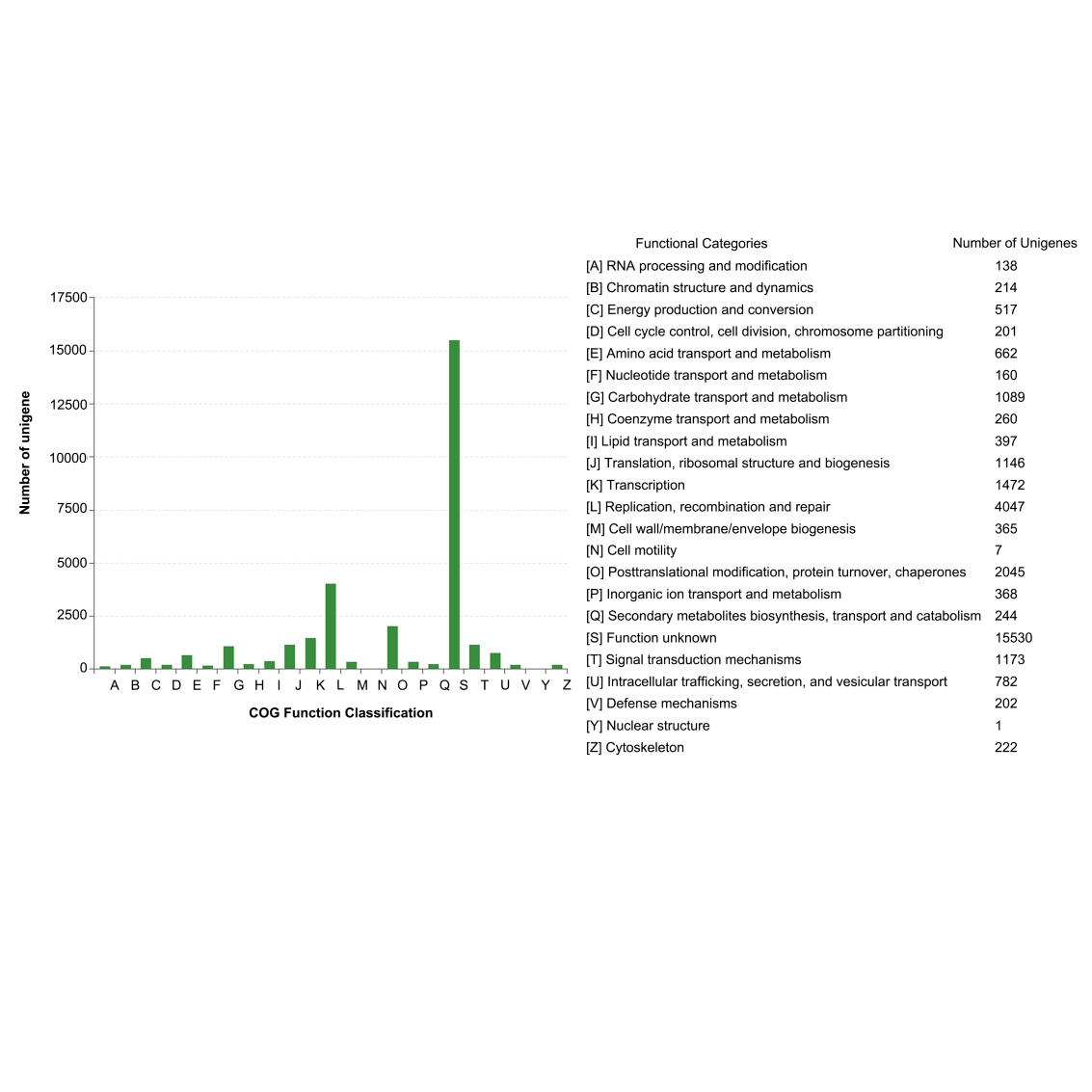


**Supplementary Figure 2.** Unigene functions were annotated by COG (Clusters of Orthologous Groups of proteins) databases. The vertical axis represents the functional classification of COG (represented by capital letters A-Z), and the horizontal axis represents the number of unigene with corresponding functions.


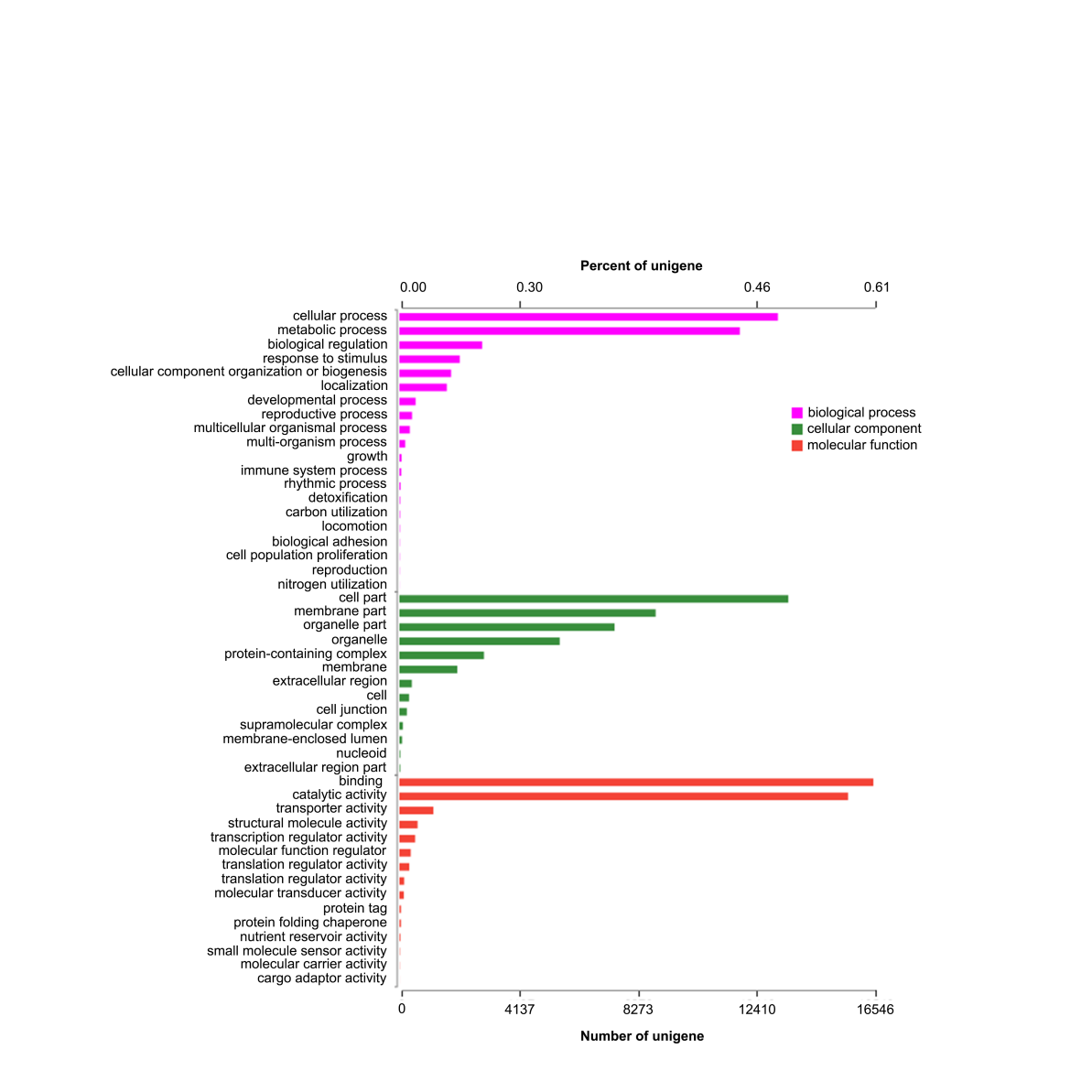


**Supplementary Figure 3.** Unigene functions were annotated by GO (Gene Ontology) databases. The vertical axis represents the classification terms of GO, the upper horizontal axis represents the percentage of unigene of corresponding GO terms in the total obtained unigene in this study, the below horizontal axis represents the number of unigene corresponding GO terms, and the three colors represent the three GO categories (BP, CC, MF).

**
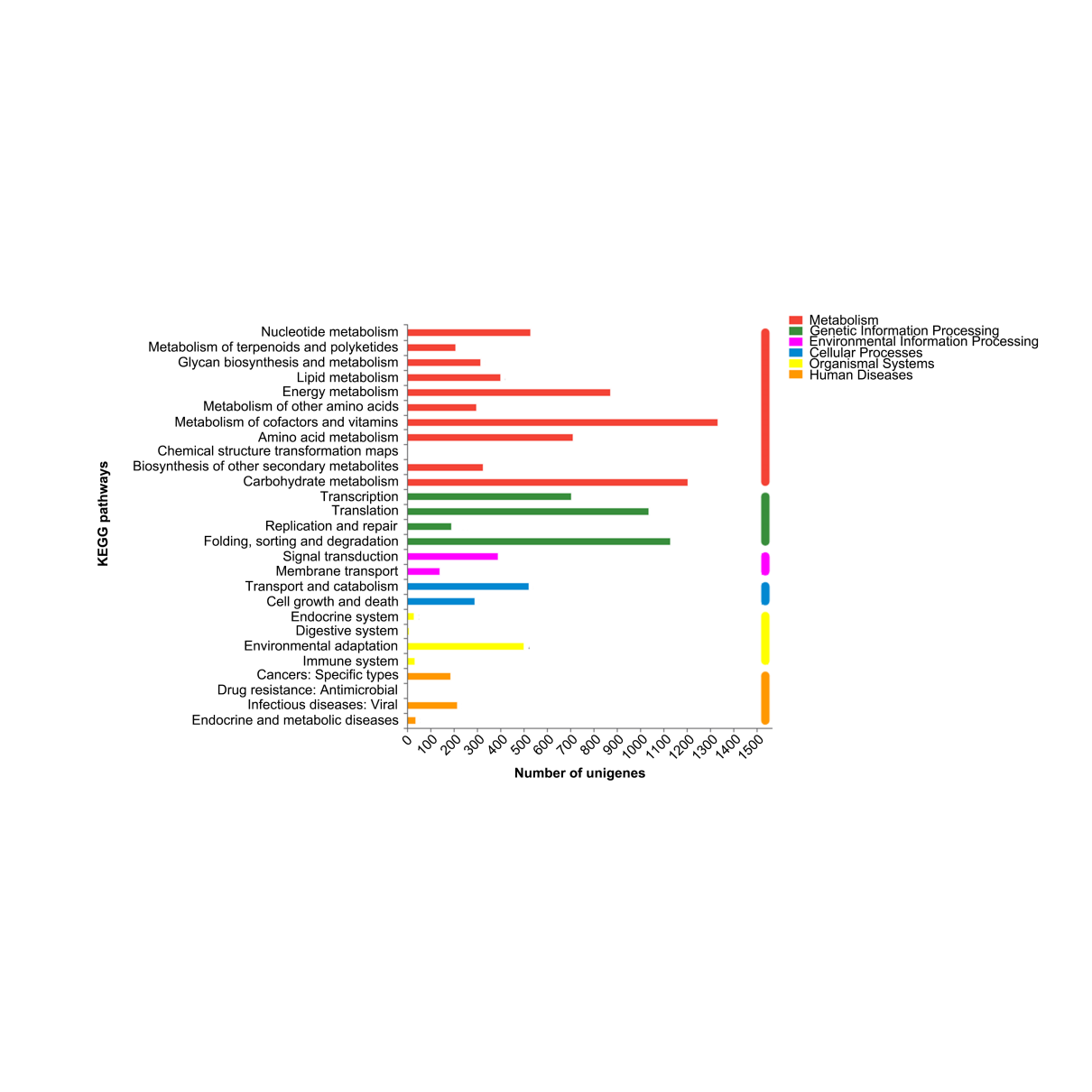
**

**Supplementary Figure 4.** Unigene functions were annotated by KEGG (Kyoto Encyclopedia of Genes and Genomes) databases. The vertical axis represents the name of KEGG metabolic pathway, and the horizontal axis represents the number of unigenes annotated to the pathway. Different colors represent different KEGG pathway categories.


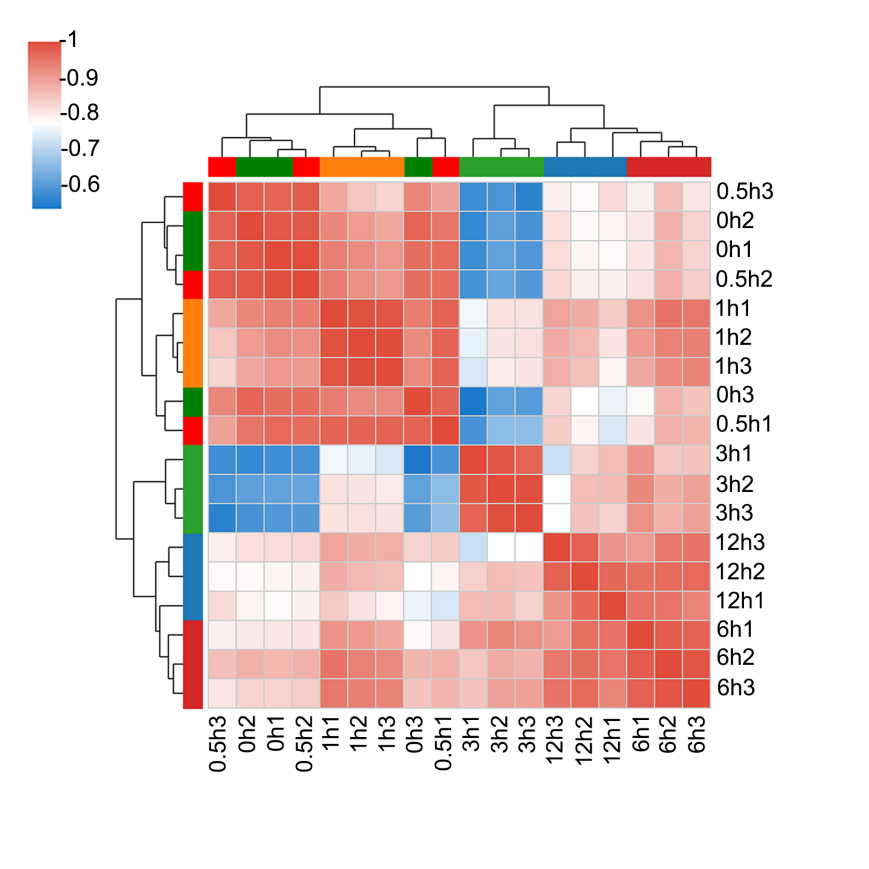


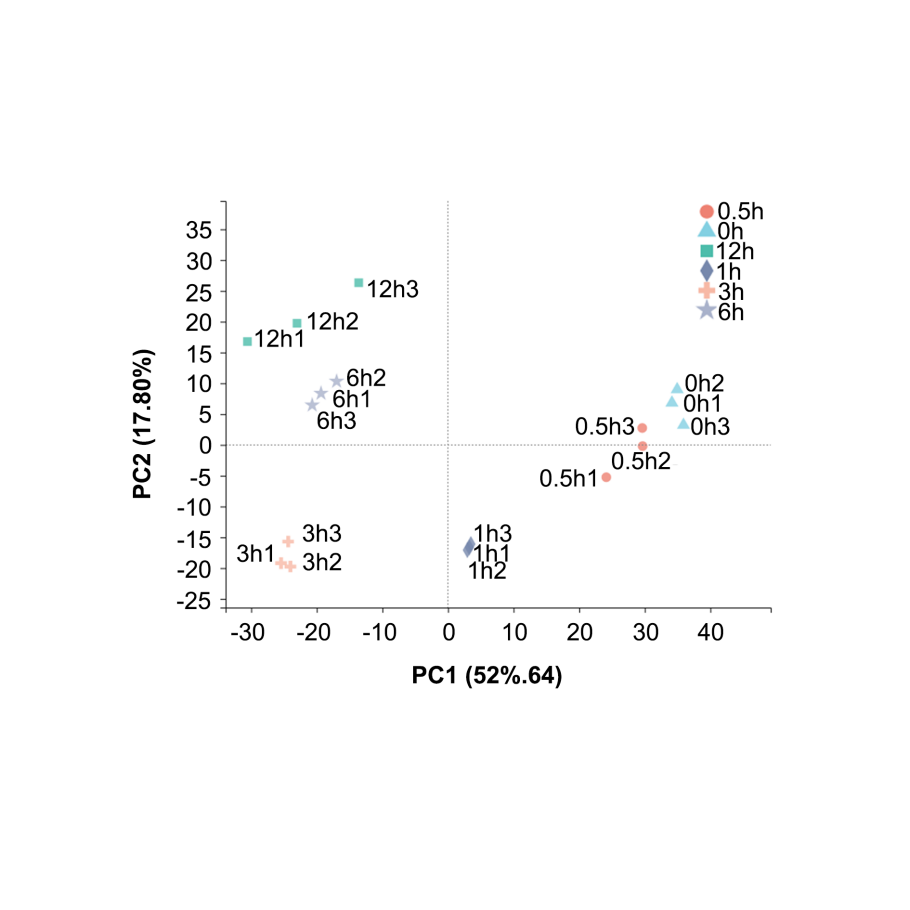
**Supplementary Figure 5.** Heat map of correlation among biological replicates for each treatment. The right and lower sides are sample names, the left and upper sides are sample clustering, and different colors represent the correlation coefficient between samples corresponding to that scale in the upper left. The closer to 1 the correlation coefficient is, the closer transcriptome expression profiles between samples are.

**Supplementary Figure 6.** Principal component analysis (PCA) among biological replicates for each treatment. Note: The distance of each point represents the distance between the corresponding samples. The closer the distance is, the higher the similarity between samples is. The vertical axis represents the contribution of a principal component N (PCN) to the differentiated samples and the horizontal axis represents the contribution of a principal component M (PCM) to the differentiated samples.

**
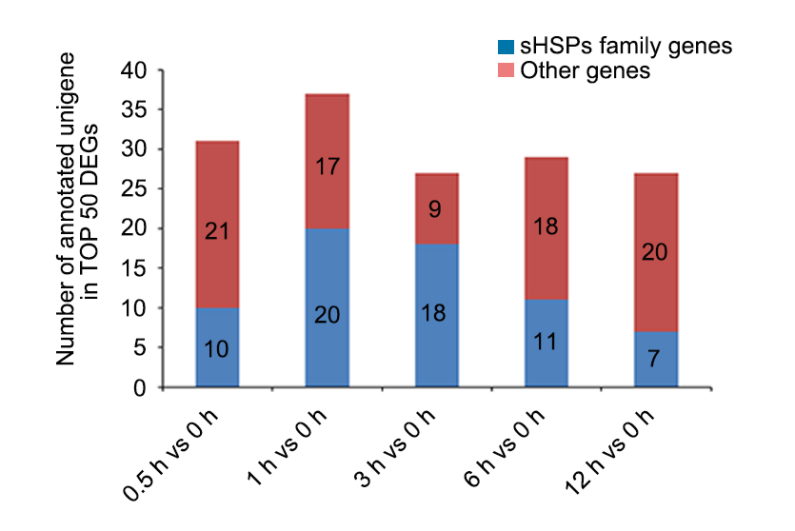
**

**Supplementary Figure 7.** The distribution of small heat shock protein family genes in the top up-regulated 50 DEGs of different heat treatment groups. Note: The red column represents the number of up-regulated small heat shock protein family genes, and the blue column represents the number of non-small heat shock protein family genes. Only the annotated genes in the top up-regulated 50 DEGs in each group are counted, so the total number of differentially expressed genes in red column and blue column in each group is less than 50.


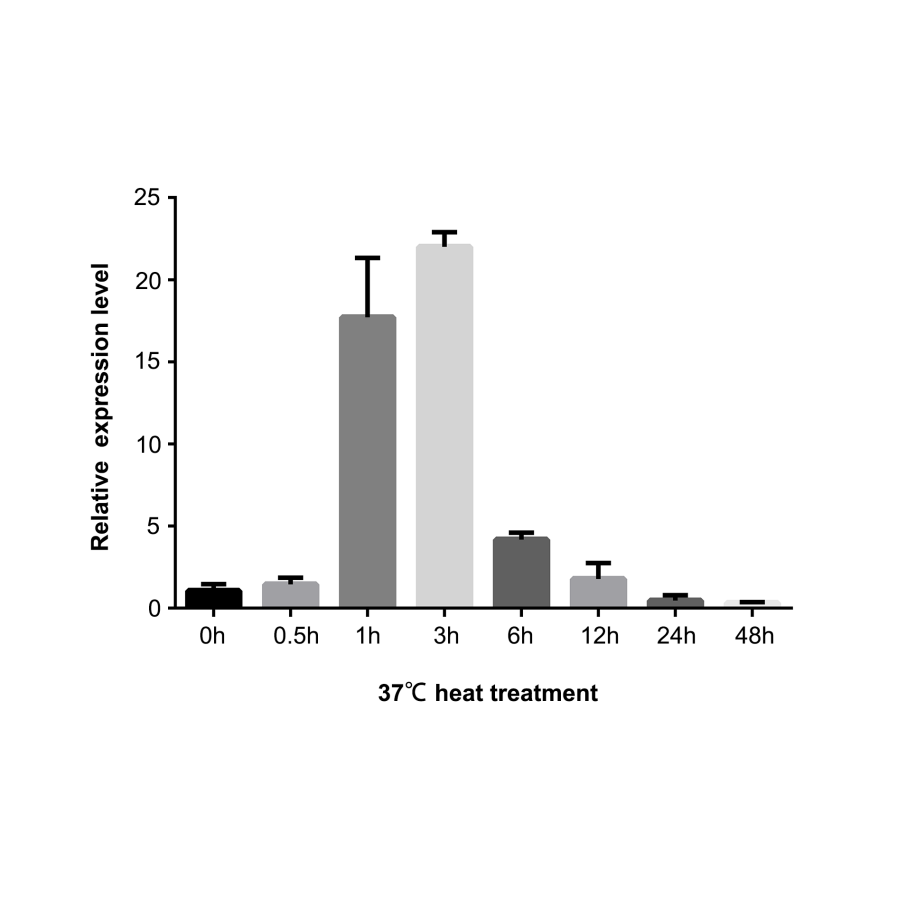


**Supplementary Figure 8.** The relative expression levels of *LlHSFA2* in lily leaves exposed to different heat stress treatments (0 h, 0.5 h, 1 h, 3 h, 6 h, 12 h, 24 h and 48 h).The aseptically grown seedlings of L. *longiflorum* ‘White Heaven’ cultured for 37 days (with 4-6 leaves and approximately 1 cm bulb diameter) were used as the materials here. The lily housekeeping gene, *18S rRNA* gene, was used as an internal control for normalization in qRT-PCR and relative gene expression levels were calculated using the 2^−ΔΔCT^ method. Data are means ± SD of three biological replicates.

**Supplementary Table1.Quality control and assembly assessment of sequencing data**

| Sample | Raw reads | Clean reads | Mapped reads | Mapped ratio | Clean bases (Mb) | Q20(%) | Q30(%) |
| --- | --- | --- | --- | --- | --- | --- | --- |
| H0h1 | 116887450 | 116339222(99.53%) | 95188540 | 81.82% | 17408.16263 | 96.61 | 91.08 |
| H0h2 | 126105462 | 125520518(99.54%) | 102180172 | 81.41% | 18789.84049 | 96.42 | 90.67 |
| H0h3 | 132363882 | 131625660(99.44%) | 107201664 | 81.44% | 19703.06328 | 95.96 | 89.79 |
| H0_5h1 | 150435804 | 149704336(99.51%) | 120736866 | 80.65% | 22411.18855 | 96.3 | 90.46 |
| H0_5h2 | 129743080 | 129220050(99.60%) | 104851214 | 81.14% | 19345.28136 | 96.69 | 91.24 |
| H0_5h3 | 147145088 | 146548102(99.59%) | 120691370 | 82.36% | 21942.48261 | 96.52 | 90.65 |
| H1h1 | 147510610 | 146876274(99.57%) | 118164026 | 80.45% | 21990.41231 | 96.41 | 90.65 |
| H1h2 | 143969014 | 143321206(99.55%) | 116857372 | 81.54% | 21455.4516 | 96.46 | 90.77 |
| H1h3 | 135295570 | 134754840(99.60%) | 108658878 | 80.63% | 20174.50083 | 96.75 | 91.35 |
| H3h1 | 169708770 | 168999892(99.58%) | 135270198 | 80.04% | 25299.56738 | 96.55 | 90.92 |
| H3h2 | 126833584 | 126323316(99.60%) | 102315822 | 81.00% | 18909.89929 | 96.75 | 91.34 |
| H3h3 | 129281914 | 128677026(99.53%) | 102555984 | 79.70% | 19258.75565 | 96.33 | 90.47 |
| H6h1 | 132222636 | 131530080(99.48%) | 107955360 | 82.08% | 19686.28566 | 96.32 | 90.49 |
| H6h2 | 136114110 | 135584364(99.61%) | 109818758 | 81.00% | 20295.15249 | 96.79 | 91.42 |
| H6h3 | 130762020 | 130145738(99.53%) | 106591048 | 81.90% | 19479.22815 | 96.35 | 90.53 |
| H12h1 | 119056254 | 118354846(99.41%) | 97163356 | 82.09% | 17709.40213 | 95.83 | 89.52 |
| H12h2 | 172328018 | 171487430(99.51%) | 141847384 | 82.72% | 25663.67907 | 96.2 | 90.19 |
| H12h3 | 109816168 | 109364486(99.59%) | 91525144 | 83.69% | 16367.06279 | 96.47 | 90.72 |

**Supplementary Table 2. Evaluation of assembly and distribution of unigene length**

| **A** | | | **B** | | |
| --- | --- | --- | --- | --- | --- |
| Type | Transcript | Unigene | Length of Unigene | Number of Unigene | Percent of Unigene |
| Total number | 96124 | 55044 | 0~500 | 19348 | 35% |
| Total base | 128788395 | 73545146 | 501~1000 | 11895 | 22% |
| Largest length (bp) | 19856 | 19856 | 1001~1500 | 6963 | 13% |
| Smallest length (bp) | 201 | 201 | 1501~2000 | 5184 | 9% |
| Average length (bp) | 1339.82 | 1336.12 | 2001~2500 | 3684 | 7% |
| N50 length (bp) | 2091 | 2249 | 2501~3000 | 2293 | 4% |
| E90N50 length (bp) | 1942 | 2194 | 3001~3500 | 1560 | 3% |
| Mean mapped percent (%) | 91.377 | 81.713 | 3501~4000 | 1165 | 2% |
| TransRate score | 0.30942 | 0.23979 | 4001~4500 | 806 | 1% |
| BUSCO score | 76.4% (2.2%) | 76.4% (2.2%) | >4500 | 2146 | 4% |
